# Future directions of forensic DNA databases

**DOI:** 10.3325/cmj.2014.55.163

**Published:** 2014-04

**Authors:** Jianye Ge, Hongyu Sun, Haiyan Li, Chao Liu, Jiangwei Yan, Bruce Budowle

**Affiliations:** 1 Institute of Applied Genetics, Department of Molecular and Medical Genetics, University of North Texas Health Science Center, Fort Worth, TX, USA; 2 Department of Forensic Medicine, Sun Yat-Sen University, Guangzhou, China; 3 Forensic Science Division, Guangdong Provincial Police Department, Guangzhou, China; 4 Guangzhou Forensic Institute, Guangzhou, China; 5 Beijing Institute of Genomics, China Academy of Sciences, Beijing, China; 6 Center of Excellence in Genomic Medicine Research (CEGMR), King Abdulaziz University, Jeddah, Saudi Arabia

Forensic DNA databases are indispensable tools of the law enforcement system. The purpose of establishing forensic DNA databases was to develop investigative leads for solving crime and usually was the purview of “criminal justice agencies for law enforcement identification purposes” (1). The forensic DNA databases of most countries generally contain two types of profiles: 1) reference profiles from convicted offenders and/or arrestee profiles; these profiles are from known sources; and 2) forensic profiles which derive from crime scenes and are characteristically from unknown sources. In a typical database search, an unknown forensic profile is searched against the convicted offender and arrestee profiles (or can be searched against other unknown forensic profiles) to determine if an association, often called a match or hit, can be found. The hit can be used to develop investigation leads. As of May 2013, China and the United States (US) maintain the two largest forensic DNA databases, containing more than 20 and 12 million profiles and have produced over 410 000 (2) and 185 000 hits (3), respectively. In addition to direct matching between known and unknown sample profiles, profiles from missing persons and their relatives, as well as unidentified human remains, are included in a number of databases (2,3). Missing person identification also is an invaluable module for investigating certain crimes. For example, as of June 2013, China has successfully identified and rescued 2455 trafficked children through the use of its DNA database (2). The US National Missing and Unidentified Persons System (NamUs), which uses other meta-data has successfully solved 3499 missing persons cases as of August 2012 (4).

As expected with the great success of the use of forensic DNA databases, new challenges are emerging. The databases are experiencing rapid growth, and thus there is a potential of increased adventitious hits; the power for current and new applications (eg, missing person identification and familial searching) require additional infrastructure support; and there is an increased desire for international data sharing (5-7), which possibly could be hampered if only a relatively small number of loci is shared among laboratories worldwide.

## Core genetic marker strategies

To rise to the challenges, two different directions or strategies have been proposed for enhancing search capabilities, the implementation of which is on-going. The Federal Bureau of Investigation (FBI) in the US has proposed to add more autosomal short tandem repeat (STR) loci to its current core set of loci (6,8). The additional loci mainly derive from those that the European system has chosen to supplement its core loci. These loci would certainly assist with international data sharing, primarily with Europe, to reduce the likelihood of adventitious matches in database searches, and to increase discriminating power for missing person identifications. However, on a practical level, little efficiency is gained with this direction, since current common commercial kits provide sufficient power for typical single-source profiles in database searches within a country, as well as with international data sharing (7). It is important to note that most databases do not add forensic unknown samples that are mixtures; they typically deconvolve the mixture, limit to only a portion of a mixture, or do not allow mixtures to be uploaded to the database. Therefore, additional autosomal loci are not likely to improve searching capabilities of detecting contributors in the database. However, kinship analyses applications (eg, missing person identification and familial searching) (7) do need to be enhanced to become more effective. One Y chromosome STR marker (ie, DYS391) was added into the new core loci described by the FBI, but the purpose was to “confirm Amelogenin null values sometimes present in DNA typing” (6). In contrast, the Amelogenin locus usually is not used in a database search. With a low dropout rate of Amelogenin-Y (eg, 0.0227% in Chinese population) (9), adding one Y-STR with relatively low discriminating power may not be the best decision to support forensic investigation leads developed through database searches.

The China Ministry of Public Security (MPS), instead, chose to establish a national Y-STR database appending to the current autosomal STR-based database. Although the decision on the core Y-STR loci of the national database is still in progress, several provincial crime laboratories already have started their own Y-STR databases with various commercially available Y-STR kits. It is well established that most violent crimes are committed by men (eg, nearly 99% of the forcible rapes, 88% of the robberies, and 85% of the burglaries are committed by men, and 88.8% homicide offenders were male) (10,11). As expected, the majority of the DNA profiles in DNA databases belong to men (eg, 87% men in Texas State DNA Index System (SDIS) in the US; 86% men in Guangdong Provincial DNA Database in China). In this sense, Y chromosome STRs, with their feature of paternal lineage, can be extremely useful in a number of forensic applications. Butler (12), a representative of the National Institute of Standards and Technology (NIST), has argued that the use of Y STRs, as proposed by Ge et al (7) and advocated by China, is not a good approach. He opined that, for example, for three of the US States, California, Illinois, and Virginia, approximately 16%-22% of the reference profiles in their DNA databases were female. Thus, adding Y STRs would provide no additional value with these profiles and searches. Although Butler ignores the fact that the overwhelming majority of profiles are from men and even greater percentage of men commit violent crimes, there is another factor to consider. An important factor driving the decision should be the gender distribution of the hits that arise in current database searches, especially of violent crimes. Since current systems search on autosomal markers, the results should not be biased by gender. Yet, anecdotally, the hits are mostly with male profiles for violent crimes. To make informed decisions on proposing the value or lack of value of marker types, the FBI and NIST should acquire the data on gender and hits before advocating an enhanced core set of markers. It should be noted that Butler (12) did rightly point out that gender proportions were different for missing persons and human remains, ie, that women make up almost half of the missing persons cases. However, the missing persons cases make up a very small portion of all cases and can be addressed with separate approaches, as has been practiced since the inception of DNA typing and missing person identification (which, for example, employs mitochondrial DNA as an additional marker).

A few Y-STRs together with autosomal STRs can provide a higher expected likelihood ratio in kinship analyses than only autosomal STRs (7) and still maintain a sufficient discrimination power of direct comparisons of single source profiles (eg, database searches and international data sharing) (7). Second, for missing person cases, distant male relatives (eg, cousins, uncles, etc) can be good references with their lineage-based Y-STRs (as would mitochondrial DNA for distant female lineage relationships), while their autosomal STR loci are very limited in their ability when only distant relatives are available to serve as reference samples (13). Third, the efficiency of familial searching can be improved with an increased number of Y-STRs, which will dramatically reduce adventitious hits due to the low accuracy afforded by current familial searching based solely on autosomal STR markers and in turn reduce unnecessary investigation and intrusions (14-16). Fourth, evidence from sexual assault cases, which often comprises more than 50% of the biological evidence submitted to forensic casework laboratories in US and China (17), tends to be mixtures of female and male DNA. Y-STR typing can quickly exclude the majority of the potential male donors especially if there is only one male contributor. Even with multiple male contributors, it can be easier to determine the number of contributors with Y-STR typing than with autosomal STR typing. Therefore, Y-STR typing has been included in the standard protocol for sexual assault cases in many Chinese crime laboratories. Fifth, in geographical areas with limited immigration, such as in China, a Y-STR profile can be used in a familial search and efficiently assist in searching for perpetrators by first screening families’ Y-STR haplotype(s) and then investigating the individuals of the Y haplotype matched family. One very successful example is the local Y-STR database developed by Zhengzhou Police Department in China. Zhengzhou, the capital of Henan Province in China, has about 4 million residents. The Zhengzhou Y-STR database was established by compiling the pedigrees of the entire city and collecting a few male samples from each male lineage. Dozens of cases have been solved in an expeditious manner with the use of this Y-STR database (2). Several other provincial or city police departments in China are following this successful strategy and are or have set up their own local Y-STR databases. Generally, over one thousand cases in China are assisted per year by Y-STR tying (2).

## Future directions

Regardless of the strengths and weaknesses of these two database strategy directions, both options require more STR loci to provide better information so forensic investigations can be assisted. However, because of the limitation of the current Capillary Electrophoresis (CE) separation and detection technology, only up to 25 ~ 30 autosomal STR and/or Y-STR loci can be multiplexed in a single kit and analysis, which apparently indirectly limits the marker capacity of the DNA database to support forensic investigations. In addition, one of the unintended consequences of a core set of markers is that analysts opt to analyze evidence with the core markers, regardless if a different set of genetic markers would be better suited for the analysis. With this strategy there will be situations where no results will be obtained with the core markers but may have been possibly attained with different more appropriate systems. Massively Parallel Sequencing (MPS) technologies may be one solution to overcome these obstacles of being able to type only about a maximum of 30 STR loci and only typing with autosomal STRs. The MPS technologies sequence DNA in a massively parallel fashion with high coverage and high throughput of specified targets. Because of the exquisitely high throughput, a large battery of genetic markers can be analyzed simultaneously, far exceeding the capacity of the current CE system. It is entirely possible that all forensically-relevant identified autosomal STRs, such as the 24 STR loci selected by Hares (6) and beyond, a set of Y STRs and X STRs, whole mitochondrial DNA genome sequences, and human identity single nucleotide polymorphisms (comprising between 400-500 markers and much more) can be typed simultaneously. Moreover, with the high throughput capacity afforded by MPS, hundreds to thousands of different samples, which can be distinguished by barcoding, may be sequenced simultaneously. All reference profiles could be typed comprehensively by MPS for a variety of genetic marker systems and best choice analyses data from evidence samples can be compared to the database of reference profiles. In addition, the sequence-generated autosomal STR data are backward compatible with existing CE-generated autosomal STR data. The inclusion of a more comprehensive set of markers for reference samples will overlap all current databases and foster investigation leads. Eventually higher accuracies of major forensic applications can be reached, and more types of forensic investigations (eg, mixture analyses, and distant kinship relationships) would be feasible.

Currently, MPS appears sufficiently robust to type reference samples for uploading DNA profiles into databases. With the technology evolving, it is likely that in the near term MPS will be able to offer the sensitivity of detection to analyze low quantity and quality DNA samples, and will be capable of analysis of forensic casework evidence. To do so, greater engagements with government agencies, research institutes, and industries are required and should be promoted.
